# The Coming Duties of the Accoucher

**Published:** 1878-07

**Authors:** 


The Coming Duties of the Accoucher.—Prof. T. G. Thomas says: "The time is not distant when confinement cases will be treated very differently from what they are at the present day. This is a subject of the utmost importance. There is the most urgent need of a radical change in the practice of a majority of the profession, and the time is ripe for the appearance of a stirring and abler paper on "The Proper Management of Natural Labor," which will awaken medical men to a sense of their duty in obstetrical cases. The physician should be expected and required to visit his patient from time to time all through her pregnancy, in order to see that every thing is progressing favorably for a successful delivery, and to remove, if possible, any condition (as albuminuria, for instance,) which is likely to interfere with this; and I am fully convinced that it will not be long before the accoucheur who does does not pursue this plan will be held culpable. Again, he will be held equally culpable if he discharge his patient at the ninth day, or at the end of a fortnight, without a physical examination to ascertain that the parts have sustained no injury from the strain and pressure of parturition, and that the process of restoration to the normal condition is going on satisfactorily.—*Maryland Med. Jour.*

